# Elevated Total Serum Immunoglobulin A Levels in Patients with Suspicion for Celiac Disease

**DOI:** 10.3390/jcm12155101

**Published:** 2023-08-03

**Authors:** Twan Sia, Leeon Bacchus, Evan Cunningham, Katie Hsia, Megan Miller, Taylor Epstein, Yasmine Myftija, Albert Mousad, Yerramalla Sai Dinesh, Harika Maddisetty, Vinay Chandra, Ziqi Guo, Anya Gupta, Stephanie Johnson, Julia Logan, Emily Mawyer, Ally Scheve, Haitong Yu, John Leung

**Affiliations:** 1 Boston Specialists, 65 Harrison Ave #201, Boston, MA 02111, USA; 2 Stanford University School of Medicine, Stanford, CA 94305, USA; 3 Division of Internal Medicine, Tufts Medical Center, Boston, MA 02111, USA; 4 Tufts University School of Medicine, Boston, MA 02111, USA

**Keywords:** IgA, tissue transglutaminase-immunoglobulin A, tTG-IgA, serology, celiac

## Abstract

Patients with classic symptoms of celiac disease are often initially tested for serum tissue transglutaminase-immunoglobulin A (tTG-IgA) and total serum immunoglobulin A (IgA) levels concurrently, as IgA deficiency can lead to falsely low tTG-IgA. There are no guidelines for incidental findings of elevated total serum IgA when testing for celiac disease. In our study, we described the proportion of patients with suspicion of celiac disease who had elevated total serum IgA and the factors that may be associated with these findings. We studied the management of these patients with incidental findings of elevated total serum IgA to identify its clinical significance. To investigate, we performed a retrospective chart review of patients who underwent celiac disease serologic testing at a single clinic from January 2017 to June 2022. We reported further laboratory workup and follow-up for patients with incidental findings of elevated total serum IgA by board-certified immunologists. In our chart review, 848 patients were identified, 85 (10.0%) of whom were found to be negative for celiac disease but had elevated total serum IgA levels (median IgA 351 mg/dL, interquartile range 324–382). Out of 85 patients, 73 were further evaluated by immunologists, with 55 patients undergoing additional laboratory workup. None were diagnosed with specific immunologic conditions. Male sex was identified as associated with elevated total serum IgA findings, and constipation was found in a statistically significant greater frequency of patients with normal total serum IgA rather than elevated total serum IgA. To provide external validation of our findings, we created a second patient cohort within the Stanford Research Repository database. Out of 33,875 patients identified, a similarly high proportion of patients were negative for celiac disease but had elevated total serum IgA levels (9.3%, 3140 patients). In this separate patient cohort, male sex was also identified as being associated with elevated total serum IgA. Our study also provides preliminary evidence that patients with incidental findings of elevated total serum IgA may not need further management or workup, as these abnormalities may not be clinically relevant without other clinical suspicions.

## 1. Introduction

Celiac disease (CD) is an autoimmune disease in which the ingestion of gluten triggers intestinal symptoms, such as abdominal pain, bloating, and bowel movement irregularities, as well as extraintestinal symptoms [1]. According to various scientific and medical societies, in patients presenting with classic symptoms of CD, such as postprandial bloating, gaseousness, and chronic diarrhea, CD must be ruled out [2,3].

The recommended diagnostic test utilized in patients with clinical suspicion for CD includes an initial serologic test for tissue transglutaminase-immunoglobulin A (tTG-IgA) while the patient is on a gluten-containing diet [1,3]. tTG-IgA is regarded as the most sensitive marker for CD diagnosis and is relatively inexpensive and easy to assay in comparison to other serologic markers [3,4]. When tTG-IgA levels are evaluated, it is important to concurrently measure total serum immunoglobulin A (IgA), as tTG-IgA can be falsely low in patients with IgA deficiency [1,3,5], which affects approximately 2% of patients with CD [5,6]. Clinical algorithms for patients with findings of IgA deficiency when being tested for CD using tTG-IgA levels suggest follow-up serological testing using tissue transglutaminase-immunoglobulin G and/or deaminated gliadin peptide immunoglobulin G, followed by duodenal biopsies [1,3].

In contrast, there is a relative lack of research on patients with findings of elevated total serum IgA when undergoing CD diagnostic testing. Previous works have suggested that total serum IgA levels can be influenced by many factors, including stress [7], diet [8,9,10], and lifestyle metrics [11,12]. To our knowledge, there are currently no studies that have described the proportion of patients who have incidental findings of elevated total serum IgA upon CD diagnostic testing [13]. Additionally, there is a notable lack of clinical guidelines for managing findings of elevated total serum IgA during evaluation for CD [1]. 

Elevated total serum IgA is found in a variety of inflammatory disorders, including IgA nephropathy [14], Henoch-Schoenlein purpura [15], chronic spinal cord injury [16], and alcoholic cirrhosis [17]. Elevated total serum IgA can also occur in conjunction with the elevation of all immunoglobulins in gammopathies, such as multiple myeloma [18], or alone in monoclonal gammopathies of undetermined significance [19]. In the absence of suspicion for specific rheumatologic, inflammatory, or polyclonal diseases, there is no putatively standard immunologic workup for elevated total serum IgA. 

Therefore, we investigated the clinical significance of elevated total serum IgA in patients with gastrointestinal complaints who were investigated for CD. In our study, we had the following aims: (1) report the proportion of patients with incidental findings of elevated total serum IgA levels in CD diagnostic testing; (2) investigate if age, sex, and symptoms are associated with normal, elevated, or low total serum IgA findings; and (3) identify outcomes of immunology evaluation and describe the clinical course of these patients during follow-up. To do this, we first investigated using a retrospective chart review at a single medical center located in Boston, Massachusetts. Then, we validated some of our findings using a retrospective database from an academic center located in the San Francisco Bay Area.

## 2. Materials and Methods

### 2.1. Patient Cohort

We initially conducted a retrospective chart review at a single medical clinic serving patients primarily from the northeastern region of the United States (hereinafter referred to as the B-S cohort [Boston Specialists cohort]). The electronic medical record was searched for all patients that had “Celiac Disease Comprehensive Panel” or “Tissue Transglutaminase Ab, IgA” and “Immunoglobulin A” ordered from Quest Diagnostics between January 2017 and June 2022. Out of 910 identified patients who underwent these laboratory tests, patients were excluded for the following reasons: (1) they were previously diagnosed with CD at a different provider, as these labs were for monitoring instead of diagnosis; and (2) if patients had labs ordered but results were not available due to technical errors. Laboratory tests were performed by Quest Diagnostics at various locations in the northeastern region of the United States. Patients with elevated total serum IgA according to the reference range (Quest Diagnostics) [20] were referred to board-certified immunologists for evaluation and follow-up. This study was deemed exempt from institutional review board approval by WCG IRB, thus the need for consent was waived.

### 2.2. Data Abstraction 

Patient demographics, laboratory results, clinical symptoms, and follow-up duration for the B-S cohort were abstracted from the electronic medical record by trained investigators. At least two investigators independently abstracted data, and a third investigator resolved any conflicting or ambiguous data. Abstractors were blinded to our study hypothesis and were not involved in the analysis or interpretation of the data. Following data abstraction, all data was deidentified, and authors were unable to identify individual patients. 

### 2.3. Statistical Analysis

Demographics and clinical characteristics of the B-S cohort patients were summarized using descriptive statistics. To identify factors associated with different total serum IgA levels, various comparisons were made among the following patient groups: patients who tested negative for CD by tTG-IgA and had total serum IgA within normal limits, patients who tested negative for CD by tTG-IgA with low total serum IgA, and patients who tested negative for CD by tTG-IgA with elevated total serum IgA. The Shapiro–Wilk test was used to determine if total serum IgA levels in our study cohort were normally distributed or not. The median ages among these patient groups were compared using the Kruskal–Wallis test. A nonparametric Kruskal–Wallis test was used instead of a one-way ANOVA due to the inability to assume the normal distribution of the analyzed population. The proportion of male patients, pediatric patients, and reported gastrointestinal and extraintestinal symptoms were compared between the patient groups using a Fisher exact statistic. This test was used rather than a chi-square test since more than one expected value in contingency tables for several investigated dependent variables was less than 5. Due to the large number of investigated factors without specific hypotheses (3 demographic and 27 clinical variables), a correction was applied to the a priori α level to minimize the false positive rate (Type I error) due to multiple comparisons across our family of null hypotheses. Using a Bonferroni correction, our significance level was determined to be α = 0.05/30 = 0.0017.

For statistically significant findings, a post-hoc pairwise comparison was conducted to identify where the specific statistically significant difference between groups was located. In this subanalysis, to correct for experiment-wise Type 1 error due to multiple comparisons for a single null hypothesis, a post-hoc Bonferroni correction was applied. The significance level was determined to be α = 0.05/3 = 0.017. For statistically significant differences identified between two specific groups, odds ratios were calculated to quantify the strength of association. Descriptive and inferential statistics were conducted using SPSS^TM^ 28.0.1.1 software (IBM Corp. (Armonk, NY, USA)).

### 2.4. External Validation Using the Stanford Research Repository

For external validation of our results using a second patient cohort, we used the Cohort Discovery tool in the Stanford Research Repository (STARR) database to look for patients who had been seen at Stanford Hospital and Clinics from 2016 to 2023 (hereinafter referred to as the STARR cohort). We searched for patients who were tested for serum tTG-IgA and total serum IgA. The STARR cohort was divided into patients who were positive for CD based on tTG-IgA, patients negative for CD based on tTG-IgA who had normal total serum IgA levels, patients negative for CD based on tTG-IgA who had low total serum IgA levels, and patients negative for CD based on tTG-IgA who had elevated total serum IgA levels. The Stanford Clinical Laboratory reference range for total serum IgA is as follows: 1 month, <52.0 mg/dL; 2 months, <47.0 mg/dL; 3 months, <46.0 mg/dL; 4 months, <72.0 mg/dL; 5 months, <83.0 mg/dL; 6 months, <67.0 mg/dL; 7–9 months, 11.0–89.0 mg/dL; 10–11 months, 16.0–83.0 mg/dL; 1 year, 14.0–105.0 mg/dL; 2 years, 14.0–122.0 mg/dL; 3 years, 22.0–157.0 mg/dL; 4–5 years, 25.0–152.0 mg/dL; 6–8 years, 33.0–200.0 mg/dL; 9–10 years, 45.0–234.0; 11+ years, 69.0–309.0. 

We abstracted sex data from the STARR Cohort Discovery tool. Age and clinical symptom data were not available as continuous variables. Per the data usage agreement, only aggregate data (rounded to the nearest 5) was available, which precluded certain types of analyses, such as regression analyses. A chi-square test was used to compare the proportion of male patients between patients who had normal total serum IgA and those with high total serum IgA. Odds ratios were then used to quantify the strength of association. No institutional board review approval was needed to use the STARR Cohort Discovery tool, as only aggregate data was used. 

## 3. Results

### 3.1. Study Cohort Characteristics

**Figure 1:**
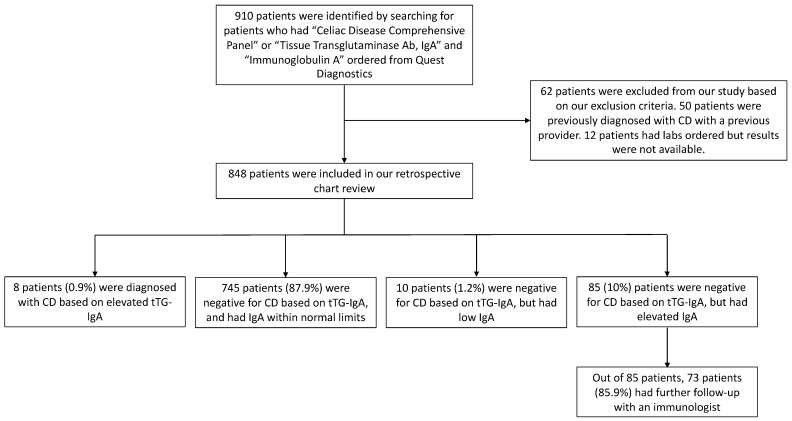
Flowchart of B-S cohort patients included in the study based on our inclusion and exclusion criteria.

To investigate, we first assembled the B-S cohort. From the electronic medical record, 910 patients were identified as having “Celiac Disease Comprehensive Panel” or “Tissue Transglutaminase Ab, IgA” and “Immunoglobulin A” ordered from Quest Diagnostics between January 2017 and June 2022. Based on our exclusion criteria, 62 patients were excluded from our analysis (Figure 1). The included 848 patients who underwent CD diagnostic testing had a median age of 28.3 years old (interquartile range {IQR}, 23.9–33.7), 32 patients (3.8%) were pediatric, and 327 patients (38.6%; Table 1) were male. Patients presented with various gastrointestinal and extraintestinal symptoms. The most common gastrointestinal symptoms were abdominal pain (480 patients, 56.6%), bloating (421 patients, 49.6%), and diarrhea (356 patients, 42%; Table 2). Extraintestinal symptoms were also noted (Table 2).

### 3.2. Proportion of Symptomatic Patients Tested for CD with Elevated Total Serum IgA

**Figure 2:**
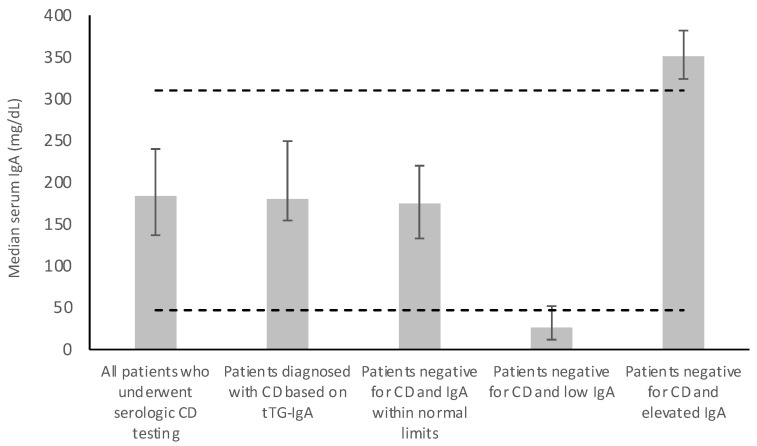
Total serum IgA of B-S cohort patients who underwent CD testing, patients diagnosed with CD, patients with IgA within normal limits, patients with low IgA, and patients with elevated IgA. Error bars represent the interquartile range. Dashed horizontal lines represent the upper and lower limits for total serum IgA for patients aged 17–60 years old, according to Quest Diagnostics.

Based on laboratory findings in the 848 included patients who underwent CD diagnostic testing, 8 patients (0.9%; Figure 1) were determined to be positive for CD based on serum tTG-IgA (tTG-IgA ≥ 15.0 U/mL) [21]. Out of 848 patients, 745 (87.9%) were negative for CD based on tTG-IgA and had total serum IgA within normal limits (median IgA 175 mg/dL, IQR 133–220), 10 (1.2%) were negative for CD based on tTG-IgA and had low total serum IgA (median IgA 26 mg/dL, IQR 11.8–52), and 85 (10%) were negative for CD based on tTG-IgA and had elevated total serum IgA (median IgA 351 mg/dL, IQR 324–382; Figure 1 and Figure 2). 

For the 85 patients (10%; Figure 1) with laboratory findings of elevated total serum IgA without diagnoses of CD, the median age was 29.6 years old (IQR 26.7–35), 4 patients (4.8%) were pediatric, and 50 patients (58.8%; Table 1) were male. This group had various gastrointestinal symptoms, including abdominal pain (39 patients, 45.9%), bloating (40 patients, 47.1%), diarrhea (39 patients, 45.9%), constipation (5 patients, 5.9%), rectal bleeding (7 patients, 8.2%), rectal pain (2 patients, 2.4%), vomiting (4 patients, 4.7%), and dysphagia (2 patients, 2.4%; Table 2). Various extraintestinal symptoms were also noted (Table 2). 

### 3.3. Association of Patient Demographics and Clinical Characteristics with Total Serum IgA Levels

For patients negative for CD based on tTG-IgA, we investigated if demographic metrics or clinical presentation were associated with total serum IgA levels (Table 1 and Table 2). A Shapiro–Wilk test was performed and showed that the distribution of total serum IgA levels (*n* = 848) departed significantly from normality (W = 0.949, *p* < 0.001), thus statistical analysis was conducted using nonparametric tests. Comparisons between patients with IgA within normal limits, patients with elevated IgA, and patients with low IgA were not significantly different in terms of median age or proportion of pediatric patients after a post-hoc Bonferroni correction was applied (*p* = 0.029, 0.274; Table 1). Statistically significant differences were found when comparing the proportion of male patients between groups (*p* < 0.001; Table 1). Among those who tested negative for CD, those with elevated total serum IgA levels were 2.5 times (reciprocal odds ratio = 0.4) more likely to be male than those with normal total serum IgA levels (Table 3A). Comparing these groups for various symptoms found constipation to be the only statistically significant finding after a Bonferroni correction (Table 2). Here, those with normal total serum IgA levels were 4.59 times more likely to report experiencing constipation than those with elevated total serum IgA levels (Table 3B).

### 3.4. Outcomes of Immunology Evaluation and Follow-Up in Patients with Elevated Total Serum IgA

All patients with incidental findings of elevated total serum IgA were recommended for follow-up with a board-certified immunologist. Out of 85 patients, 73 patients (85.9%; Figure 1) followed up with a board-certified immunologist for a median duration of 0.15 years (IQR 0.10–0.68) and had a median of 1 visit (IQR 0–2; Table 4). During this time, 55 patients (64.7%) had further laboratory investigation: repeated total serum IgA (7 patients, 82%), total serum immunoglobulin G (8 patients, 9.4%), total serum immunoglobulin M (8 patients, 9.4%), total serum immunoglobulin E (3 patients, 3.5%), complete blood count with differential (52 patients, 61.2%), lymphocyte subset panel (3 patients, 3.5%), erythrocyte sedimentation rate (16 patients, 18.8%), C-reactive protein (10 patients, 11.8%), total complement (48 patients, 56.5%), and serum protein electrophoresis (4 patients, 4.7%; Table 4). Despite the immunologist’s follow-up and laboratory investigation, none of the 73 patients who had elevated total serum IgA were diagnosed with an immunologic condition or had an identified etiology for elevated total serum IgA. 

### 3.5. External Validation of the Association of Male Sex and Elevated Total Serum IgA

To provide external validation of our findings in another cohort of patients from a different geographic location and clinical laboratory, we searched the STARR database for patients who were tested for serum tTG-IgA and total serum IgA. From a total of 33,875 STARR cohort patients, 1970 patients (5.8%) were diagnosed with CD based on serum tTG-IgA, 27,405 patients (80.9%) had normal total serum IgA levels, 1360 patients (4.0%) had low total serum IgA, and 3140 patients (9.3%) had high total serum IgA (Table 5). The proportion of patients who underwent CD testing and had incidental findings of elevated total serum IgA was similar between the B-S and STARR cohorts (10.0% B-S vs. 9.3% STARR).

To validate the association between male sex and elevated total serum IgA that was previously found in the B-S cohort, we investigated the proportion of male patients with normal versus high total serum IgA in the STARR cohort. We found that the proportion of male patients who were negative for CD based on tTG-IgA and had elevated total serum IgA was significantly different from the proportion of male patients who were negative for CD based on tTG-IgA and had total serum IgA within normal limits (*p* < 0.0001; Table 5). Among those who tested negative for CD, those with elevated total serum IgA levels were 1.29 times more likely to be male than those with normal total serum IgA levels (Table 5). 

We were unable to validate the association of constipation in the STARR cohort, as we could not obtain clinical signs and symptoms from the Cohort Discovery tool. We also were not able to review the patient medical records to describe the clinical course of the STARR patients with elevated total serum IgA. 

## 4. Discussion

Patients with clinical presentations of gastrointestinal symptoms such as diarrhea, abdominal pain, and bloating often have CD ruled out using serologic tests, most commonly tTG-IgA [3,4]. Since IgA deficiency can lead to false-negative results on CD diagnostic testing by serum tTG-IgA, the gold standard is to concurrently obtain total serum IgA at the time of CD testing [1,3,5]. Though there are clinical algorithms for patients with low total serum IgA levels, there are no guidelines currently available for incidental findings of elevated total serum IgA when testing for CD [1]. 

In our retrospective chart review of gastroenterology patients who were tested for CD by serum tTG-IgA level in the B-S cohort, we identified 10.0% of symptomatic patients tested for CD with incidental findings of elevated total serum IgA. Given that reference ranges in clinical chemistry are generally defined as 2.5th to 97.5th percentiles [13,22], this proportion appears to be unexpectedly large (10.0% actual compared to 2.5% expected; Figure 1). This finding was also corroborated by the high proportion of patients with elevated IgA in the STARR cohort (9.3%). It is unclear why our cohort of patients with gastroenterological complaints has a higher prevalence of elevated total serum IgA in comparison to the general population. Since stress or diet has been previously suggested to be associated with serum immunoglobulin levels [7,8,9,10,13], one possible hypothesis may be that symptomatic patients with clinical suspicion for CD may be empirically trying certain elimination diets to alleviate their gastroenterological complaints or be experiencing higher levels of stress due to their symptoms. Unfortunately, our electronic medical record did not contain consistent, reliable data regarding stress or diets, so we were not able to adjust for these variables. 

We investigated demographic and symptomatic features as predictive factors of whether patients would have normal, elevated, or low IgA levels using the B-S cohort. Using a conservative statistical approach, we found that there was a significant difference in sex in these groups, and in patients with constipation (Table 1, Table 2 and Table 3). Male patients had 2.5 times higher odds of having elevated total serum IgA versus IgA within normal limits (Table 3A). This finding was supported by the STARR cohort, in which male patients had 1.29 times higher odds of having elevated total serum IgA than IgA within normal limits, however, the strength of the association appeared to be much more modest (Table 5). Previous literature describing the effects of sex on total serum IgA suggests that male patients tend to have higher total serum IgA than female patients [13]. However, neither Quest Diagnostics nor Stanford Clinical Laboratories account for sex in their reference ranges, which may have resulted in a larger proportion of male patients in the high IgA group. 

In the B-S cohort, patients with constipation were also found to have 4.59 times the odds of having normal total serum IgA levels versus elevated levels. This finding was unable to be validated in the STARR cohort, as we were unable to obtain clinical signs and symptoms data. The mechanism for why the presence of constipation was significantly different in patients with normal, elevated, and low IgA is unclear and warrants further investigation. 

Previous work comparing total serum IgA levels in older versus younger patients reported older age to be associated with higher total serum IgA [12,13]. In the B-S cohort, we did not find any significant association between age or proportion of pediatric patients with the different total serum IgA groups, which may be due to the fact that our patient cohorts were relatively homogenous in age. Further research on this topic should seek to include patients with a wider distribution of ages. Another limitation of our study is that the groups used in our analysis of the B-S cohort have a small sample size (patients negative for CD and low total serum IgA, *n* = 10), which may reduce statistical power. We did not analyze age in the STARR cohort, as it was not available as continuous data. 

Following incidental findings of elevated total serum IgA levels, we found that despite further immunologic workup, none of the B-S cohort patients were diagnosed with immunologic conditions or had a pathological cause of elevated total serum IgA. This result suggests that elevated total serum IgA in symptomatic patients investigated for CD may not be clinically relevant without suspicion of specific rheumatologic or inflammatory diseases, and therefore, may not warrant further management. However, it is important to note that our follow-up period was relatively short (median 0.15 years, median 1 visit; Table 4). In addition, our cohort only had mildly elevated total serum IgA (median 351 mg/dL, IQR 324–382; Figure 2) in comparison to Quest Diagnostic’s upper limit of 310 mg/dL for patients aged 17–60 years old. Further research should investigate if clinical outcomes differ in patients with higher total serum IgA than in our study cohort. 

There are several other limitations to our study to consider. Our data is based only on results from Quest Diagnostics and Stanford Clinical Laboratories. Clinical laboratories vary in their reference ranges for total serum IgA, which is evident when comparing some of the major laboratories in the United States (Quest Diagnostics, Lab Corp, Mayo Clinic Laboratories, ARUP Laboratories, Spectrum Health; Table 6) [20,23,24,25,26]. Of note, reference ranges vary in age granulation, and some clinical laboratories adjust based on the patient’s sex. It is important to consider that reference ranges may not be concordant due to differences in analysis methodology (immunoturbidimetry or nephelometry), specific protocols, or equipment [27]. Therefore, each laboratory’s reference range is likely to be calibrated to its own specifications. Nevertheless, future investigations should consider a multi-site study design with data from various clinical laboratories. 

Furthermore, as a retrospective chart review, our study is limited by the data available in our electronic medical record. We were unable to extract certain characteristics from the electronic medical record, which may also be associated with elevated total serum IgA. Male sex and non-Caucasian ethnicity are associated with elevated total serum IgA. Alcohol consumption is associated with higher total serum IgA, while smoking is associated with lower total serum IgA. Certain cardiometabolic risk factors, such as hypertension and obesity, are associated with higher total serum IgA levels. Other factors that influence total serum IgA levels include lifestyle factors (psychological stress, sleep deprivation, exercise) and geography (air pollution, environmental temperature) [13]. Certain conditions with elevated total serum IgA may also influence the demographics of patients with elevated total serum IgA, such as IgA nephropathy, in which male patients have over two times higher incidence than female patients [28]. Further research should attempt to control for these confounders.

Our study is preliminary in nature and provides several possible routes for further inquiry. Future studies may investigate whether or not total serum IgA is elevated in patients with gastrointestinal complaints, as well as the mechanism behind this phenomenon. These studies may consider matching for various factors that may affect total serum IgA, such as age, sex, and lifestyle. These investigations may also benefit from a multi-site collaboration to capture the variance in total serum IgA that may be expected due to geographic differences or reporting from different clinical laboratories with disparate reference ranges and methodologies. Future investigations may also attempt to confirm whether or not elevated total serum IgA levels in patients with gastrointestinal complaints are clinically significant through long-term follow-up and standardized laboratory investigations. These studies may influence future algorithms for CD testing by informing clinicians of what to do when total serum IgA is elevated, but tTG-IgA is within normal limits. 

## Figures and Tables

**Table 1:** Demographics of B-S cohort patients who underwent serologic celiac disease diagnostic testing.

Demographics	Patients Who Had Serologic CD Testing (*n* = 848)	Patients Diagnosed with CD Based on tTG-IgA (*n* = 8)	Patients Negative for CD and IgA within Normal Limits (*n* = 745)	Patients Negative for CD and Low IgA (*n* = 10)	Patients Negative for CD and Elevated IgA (*n* = 85)	*p*-Value ^1^
Age, median (IQR)	28.3 (23.9–33.7)	37.8 (23.7–49.3)	28 (23.6–33.5)	26.2 (22.1–28.1)	29.6 (26.7–35.0)	0.029
Pediatric, *n* (%)	32 (3.8)	1 (12.5)	26 (3.5)	1 (10)	4 (4.8)	0.274
Male, *n* (%)	327 (38.6)	2 (25)	270 (36.2)	5 (50)	50 (58.8)	<0.001 *

CD, celiac disease; IgA, immunoglobulin; IQR, interquartile range; tTG-IgA, tissue transglutaminase-immunoglobulin A. ^1^ Comparisons made between the following groups: patients negative for CD and IgA within normal limits, patients negative for CD and low IgA, and patients negative for CD and elevated IgA. Comparisons of age were made using the Kruskal–Wallis test. Comparisons of the proportion of pediatric and male patients were made using a Fisher exact test. * denotes a statistically significant finding at a priori level of *p* ≤ 0.0017 after Bonferroni correction.

**Table 2:** Clinical presentation of B-S cohort patients who underwent serologic celiac disease diagnostic testing.

Symptoms	Patients Who Had Serologic CD Testing (*n* = 848)	Patients Diagnosed with CD Based on tTG-IgA (*n* = 8)	Patients Negative for CD and IgA within Normal Limits (*n* = 745)	Patients Negative for CD and Low IgA (*n* = 10)	Patients Negative for CD and Elevated IgA (*n* = 85)	*p*-Value ^1^
Symptoms, *n* (%)						
Abdominal pain	480 (56.6)	2 (25)	433 (58.1)	6 (60)	39 (45.9)	0.143
Bloating	421 (49.6)	3 (37.5)	372 (49.9)	6 (60)	40 (47.1)	0.712
Diarrhea	356 (42)	1 (12.5)	310 (41.6)	6 (60)	39 (45.9)	0.403
Constipation	173 (20.4)	1 (12.5)	166 (22.3)	1 (10)	5 (5.9)	<0.001 *
Rectal bleed	63 (7.4)	2 (25)	52 (7)	2 (20)	7 (8.2)	0.195
Rectal pain	13 (1.5)	1 (12.5)	10 (1.3)	0 (0)	2 (2.4)	0.440
Nausea	154 (18.2)	3 (37.5)	136 (18.3)	4 (40)	11 (12.9)	0.092
Vomiting	49 (5.8)	1 (12.5)	44 (5.9)	0 (0)	4 (4.7)	0.894
Dysphagia	31 (3.7)	1 (12.5)	26 (3.5)	2 (20)	2 (2.4)	0.058
Loss of appetite	29 (3.4)	0 (0)	27 (3.6)	0 (0)	2 (2.4)	0.831
Weight loss	41 (4.8)	0 (0)	32 (4.3)	1 (10)	8 (9.4)	0.065
Weight gain	6 (0.7)	0 (0)	4 (0.5)	0 (0)	2 (2.4)	0.180
Chest pain	12 (1.4)	0 (0)	10 (1.3)	0 (0)	2 (2.4)	0.440
Back pain	7 (0.8)	0 (0)	7 (0.9)	0 (0)	0 (0)	0.999
Peripheral neuropathy	24 (2.8)	0 (0)	21 (2.8)	0 (0)	3 (3.5)	0.797
Fatigue	52 (6)	3 (37.5)	39 (5.2)	1 (10)	9 (10.6)	0.088
Migraine	21 (2.5)	0 (0)	19 (2.6)	0 (0)	2 (2.4)	0.999
Brain fog	17 (2)	0 (0)	14 (1.9)	0 (0)	3 (3.5)	0.515
Dizziness	9 (1.1)	0 (0)	9 (1.2)	0 (0)	0 (0)	0.649
Anxiety	2 (0.2)	0 (0)	2 (0.3)	0 (0)	0 (0)	0.999
Insomnia	4 (0.5)	0 (0)	3 (0.4)	0 (0)	1 (1.2)	0.382
Anemia	52 (6)	3 (37.5)	20 (2.7)	0 (0)	2 (2.4)	0.999
Edema	6 (0.7)	0 (0)	6 (0.8)	0 (0)	0 (0)	0.999
Dermatitis	52 (6)	1 (12.5)	47 (6.3)	1 (10)	3 (3.5)	0.403
Urticaria	50 (5.9)	0 (0)	45 (6)	0 (0)	5 (5.9)	0.999
Pruritus	12 (1.4)	0 (0)	11 (0.1)	0 (0)	1 (1.2)	0.999
Other atopic conditions	193 (22.8)	2 (25)	162 (21.7)	1 (10)	28 (32.9)	0.046

CD, celiac disease; IgA, immunoglobulin; tTG-IgA, tissue transglutaminase-immunoglobulin A. ^1^ Comparisons made between the following groups: patients negative for CD and IgA within normal limits, patients negative for CD and low IgA, and patients negative for CD and elevated IgA. Comparisons were made using a Fisher’s exact test. * denotes a statistically significant finding at a priori level of *p* ≤ 0.0017 after Bonferroni correction.

**Table 3:** Post-hoc pairwise comparison of (A) male sex and (B) constipation in B-S cohort patients negative for celiac disease based on tissue transglutaminase-immunoglobulin A.

A. Male sex in patients negative for celiac disease.	
Comparative Groups	*p*-value
Normal versus elevated immunoglobulin A ^1^	<0.001 *
Normal versus low immunoglobulin A	0.279
Elevated versus low immunoglobulin A	0.417
B. Constipation in patients negative for celiac disease.	
Comparative Groups	*p*-value
Normal versus elevated immunoglobulin A ^2^	<0.001 *
Normal versus low immunoglobulin A	0.313
Elevated versus low immunoglobulin A	0.497

In (A) ^1^ odds ratio = 0.40 (95% CI: 0.25, 0.63) * denotes a statistically significant finding using Fisher’s exact test at a priori level of *p* ≤ 0.05/3 = 0.017 after Bonferroni correction for multiple comparisons. In (B) ^2^ odds ratio = 4.59 (95% CI: 1.83, 11.51) * denotes a statistically significant finding using Fisher’s exact test at a priori level of *p* ≤ 0.05/3 = 0.017 after Bonferroni correction for multiple comparisons.

**Table 4:** Immunologist follow-up and workup for B-S cohort patients with incidental findings of elevated serum immunoglobulin A.

Immunologist Follow Up	Patients Negative for Celiac Disease and Elevated Immunoglobulin A
Patients with ≥1 follow up	73 (85.9)
Number of follow-ups, median (IQR)	1 (0–2)
Follow-up duration, median years (IQR)	0.2 (0.1–0.7)
Patients completing additional laboratory workup, *n* (%)	55 (64.7)
Laboratory workup, *n* (%)	
Immunoglobulin A, repeated	7 (8.2)
Immunoglobulin G	8 (9.4)
Immunoglobulin M	8 (9.4)
Immunoglobulin E	3 (3.5)
Complete blood count with differential	52 (61.2)
Lymphocyte subset panel	3 (3.5)
Erythrocyte sedimentation rate	16 (18.8)
C-reactive protein	10 (11.8)
Complement, total	48 (56.5)
Serum protein electrophoresis	4 (4.7)

**Table 5:** Sex of STARR cohort patients who were tested for tTG-IgA and total serum IgA.

Characteristics	Patients Diagnosed with CD Based on tTG-IgA	Patients Negative for CD and IgA within Normal Limits	Patients Negative for CD and Low IgA	Patients Negative for CD and High IgA	*p*-Value
*n*	1970	27,405	1360	3140	
Male, *n* (%) ^1^	775 (39.3)	10,595 (38.7)	605 (44.4)	1410 (44.9)	<0.0001 *

^1^ Odds ratio = 1.29 (95% CI: 1.20, 1.39) * denotes a statistically significant finding. Comparisons made between patients negative for CD and IgA within normal limits and patients negative for CD and elevated IgA using a chi-square test.

**Table 6:** Comparison of serum immunoglobulin A range of major clinical laboratories in the United States.

Laboratory	Reference Range ^1^ (mg/dL)	Age Granulation	Sex-Based Reference Ranges	Methodology
Quest Diagnostics [20]	47–310	13 age groups	No	Immunoturbidimetry
Lab Corp [23]	87–352	7 age groups	Yes	Immunoturbidimetry
Mayo Clinic Laboratories [24]	61–356	11 age groups	No	Nephelometry
ARUP Laboratories [25]	68–408	6 age groups	No	Immunoturbidimetry
Spectrum Health [26]	60–350	6 age groups	No	Immunoturbidimetry

^1^ Reference range for 28.3-year-old female, the median age and majority sex of our study population.

## Data Availability

All relevant deidentified data, analytical methods, and study materials are stored in a HIPAA-compliant cloud-based storage, and access to these files will be provided upon request, by contacting J.L. (John Leung) at drjohnleung@bfac.org.
